# A community service doctor’s experiences of mental healthcare provision in rural Eastern Cape

**DOI:** 10.4102/safp.v66i1.5849

**Published:** 2024-03-15

**Authors:** Divan Rall, Leslie Swartz

**Affiliations:** 1 Department of Psychology, Faculty of Arts and Social Sciences, Stellenbosch University, Stellenbosch, South Africa

**Keywords:** public mental healthcare, rural mental healthcare, community service doctor, Eastern Cape, South Africa

## Abstract

**Background:**

Literature shows that in South Africa there are insufficient resources to meet mental healthcare needs. At general or district hospital level, the non-specialist doctor is often responsible for the holistic assessment and management of mental health service users. Such situations inevitably increase doctors’ care load as they are required to treat across disciplines. We highlight the particular challenges faced by a community service (CS) doctor in this context.

**Methods:**

The presented case study formed part of a larger project that investigated public mental healthcare provision in the Eastern Cape province. Data were collected through a once-off semi-structured interview with the participant. The interview was transcribed and data analysed by utilising thematic analysis to yield results.

**Results:**

The study suggests that the CS doctor experiences being overloaded with duties, and feels overwhelmed in a healthcare context that lacks resources needed for service provision, which may lead to inadequate mental healthcare provision to public health service users.

**Conclusion:**

Healthcare facilities in rural parts of the Eastern Cape province are in need of assistance. This in-depth account highlighted the consequences of working on the front line of a disadvantaged and under-resourced health system. The presented account can be interpreted as a cry for help by CS doctors for relevant authorities to improve access and provision of mental healthcare in the area.

**Contribution:**

The paper provides an exploration of the circumstances wherein mental healthcare is provided in rural parts of South Africa.

## Introduction

One way of addressing resource issues in the South African public health system has been through the introduction of the compulsory community service (CS) programme for doctors.^1^ The programme aims to ensure improved healthcare for all South Africans. It also aims to provide young doctors with an opportunity to develop their skills, facilitate professional development, and prepare them for their future careers. The majority of CS doctors between 2000 and 2014 reported having an overall positive experience during their placements. They experienced making a difference by aiding service delivery, and reported having developed professionally.^1,2^

Schierenbeck et al.^3^ highlighted that healthcare workers perceived barriers to access to mental healthcare for service users in the Eastern Cape province, and these problems appear to have persisted.^4^ However, there is a gap in literature that highlights CS doctors’ experiences of providing mental healthcare at district hospital level in South Africa. An investigation into CS doctors’ experiences in this part of the country is needed.

The Eastern Cape is one of the poorest provinces in South Africa with a high mental health service user load and limited mental health resources.^5^ We know that healthcare workers find it difficult to provide adequate care in overstretched healthcare systems.^6^ Given the aforementioned circumstances and challenges existent in the Eastern Cape, these issues may come into sharper focus in this province.

This study forms part of a broader study on mental healthcare provision in the Eastern Cape. We explored the experiences of a range of service providers (e.g., nurses, social workers, doctors, and facility managers) and of patients regarding access to and provision of public mental healthcare services at primary and secondary healthcare level.^7^ During our investigation, we became aware of the particular challenges faced by a CS doctor in this context that we wish to highlight and report on. This case study focusses on the experiences of this CS doctor, who chose the pseudonym Cottonwool.

### Research aims and objectives

The case study aims to provide a description of a CS doctor’s experiences while trying to provide mental healthcare in rural parts of the Eastern Cape. In doing so, we wish to inform the readers, our colleagues, and key role players about the challenges that newly trained generalist doctors can face in providing mental healthcare in the South African public health sector. We hope that our findings can be used to further develop and facilitate the implementation of support structures to our healthcare personnel locally and other rural areas in the country.

## Research methods

### Participant recruitment and sampling

The larger project investigated health facility managers, healthcare workers, and mental healthcare users’ (MHCUs) experiences on issues around access to and provision of public mental healthcare in the Dr Beyers Naudé Local Municipality (DBNLM) district of the Eastern Cape. Cottonwool was one of 14 healthcare workers interviewed in the larger project. Cottonwool was the only CS doctor who volunteered and was purposely selected to inform about their experiences as a young, generalist clinician who delivers mental healthcare in this setting.

### Research design, data collection, and analysis

The presented work formed part of a larger project that utilised a cross-sectional research design. During analysis of the project’s data, we noticed that Cottonwool’s experiences highlighted the multi-dimensional challenges, and consequences thereof, that front-line health workers face in providing mental healthcare in the area, and we decided to report their case in an article. A case study approach^8^ is used for purposes of this article. Data were collected from Cottonwool through a once-off semi-structured interview (please see Table 1-A1 in Appendix 1 for the interview guide) of approximately 60 min long at their place of work. The interview was conducted in English. The interview was audio-recorded, and transcribed. Themes were developed by grouping narratives that report on similar experiences or situations together by utilising thematic analysis.^9^

## Findings

### Overloaded with duty and feeling overwhelmed

Cottonwool felt overloaded, describing how they had to treat a large number of outpatients on a day. This overload takes its toll on them, and Cottonwool described how they are emotionally affected and overwhelmed by the workload:

‘I saw a ridiculous amount of patients yesterday and I was completely mentally, physically, emotionally drained when I was done yesterday. And I woke up this morning and I couldn’t, I’m not sick but I couldn’t, I didn’t feel like I want to hear anyone tell me about their chest pain, their headache, their sore foot because I don’t care. I’ve heard enough for, for the next three days.’

### Lack of access to resources needed for care provision

Cottonwool believed they did not have access to sufficient resources needed to provide care to severely ill service users that need specific interventions to facilitate their treatment at the hospital. The lack of resources exists from facility level down to treatment level. Cottonwool explained the following regarding the lack of resources required for the treatment of severe mental health conditions:

‘We don’t have restraints. We don’t have half the medication we need to sedate. We have to use midazolam instead of diazepam … Patients are here for far longer than 72 hours, because we don’t do MCS [*referring to microscopy, culture, and sensitivity urine tests*] on the weekend because our lab doesn’t do it … so they’ll come in Friday, but then those tests are done on Monday. So they actually only done within 72 hours, six, seven days into their 72 hours. [*In South African law, 72 hours is the limit that mental health service users can be admitted for prescribed involuntary care where they can be assessed, treated, and managed*].’

Cottonwool further reported:

‘… We don’t have trained mental health care nurses … the nursing staff are not equipped on how to restrain patients safely. They don’t know how to restrain so when we prescribe restraints, they don’t feel comfortable doing it.’

Cottonwool noted that lacking physical, structural, and human resources is hindering their ability to provide more comprehensive care to patients:

‘We’re lacking not only in resources in terms of medication and building and all that, but we lacking in human resource. We don’t have the people … We don’t have enough doctors. There’s [*sic*] not enough social workers. There’s [*sic*] not enough psychologists.’

### Inadequate mental healthcare provision

Cottonwool explained that mental healthcare provision is inadequate and not at the level needed for appropriate mental healthcare to service users. They explained that because of high workload and time constraints, their ability to adequately attend to and treat a patient can be restricted. As a result, the patient continues to live with an inadequately treated mental illness:

‘The answer is simple. Our mental health care users are not getting what they need to be getting. Our management of mental health care users is subpar. It is insufficient. It’s not fair to the user … A pure example, this lady that came in … She is not okay. The fact that everyone is saying she’s okay is a misnomer. Like, everyone thinks like that is her baseline. Her baseline is not right … We need to tailor her medication … No one has the time or the effort or the anything … We’re giving her Epilim and injecting her with water and saying go into the community … It’s not, it’s not okay, but it’s what we’re doing because we don’t have … a choice. Are we providing adequate care? Adequate provision? No. No time. No resources. Too many patients. The volume is too big for the amount of services we can offer.’

## Discussion

Cottonwool described that they are overwhelmed with work responsibilities at facilities that are not fully equipped with, for example separate wards or enough specialised nursing staff for appropriate mental healthcare provision. Facilities such as this district hospital in the DBNLM area do not have adequate infrastructure to house acute mentally ill patients. This places a significant strain on the facility and its healthcare workers. In line with the literature on the CS placement year,^1^ Cottonwool’s account describes how doctors are committed to making a difference for their patients. However, they are challenged by the reported hurdles in the public health sector when trying to fulfil that commitment. The challenges are so extensive that basic service delivery to patients is obstructed. The combination of high work volume and lack of resources results in the reality that service users are not receiving appropriate mental healthcare.

Cottonwool’s narrative suggests that the current state of public healthcare in this region of the country not only burdens our young doctors, but also diminishes civilians’ right to adequate mental healthcare. It leads to a scenario where doctors like Cottonwool struggle physically and mentally to meet the demands of their work. At the same time, the treatment gaps can contribute to diminished recovery and therefore returning service users, which perpetuates the burden on public healthcare facilities.

Healthcare facilities in this part of the Eastern Cape are in need of assistance. Many patients need specialised mental health assessment, treatment and management – expertise that clinicians like psychiatrists and psychologists receive many years of training for. Assistance from other professionals like occupational therapists and social workers may offer relief and has been suggested by others^10^ as key service providers at this level of care. But they also have a wide range of competing demands. Doctors cannot manage the overflow of mental health patients together with the physically ill who need their attention.

South Africa has among the most progressive legislation in the world in the form of the *Mental Health Care Act, 2002* (MHCA),^11^ and there are numerous important interventions in terms of strengthening mental health systems in the country. The MHCA, for example, instructs that MHCUs are entitled to appropriate care and rehabilitation by adequately trained clinicians or suited facilities. What Cottonwool’s heartfelt account demonstrates, however, is the gap that is experienced at a personal level between principles and policies on the one hand, and practice on the other. It makes sense logistically to give CS doctors the responsibility to provide mental healthcare as it forms part of facilitating a person’s holistic general health and well-being, but we can see from Cottonwool’s experiences that this may have unintended consequences. Their experiences suggest that they desperately want to help patients, but do not have access to resources to facilitate adequate mental healthcare. Patients with complex, acute, and serious mental illness may overwhelm young generalists like Cottonwool who have limited training of the nuances in psychiatric management and care. Cottonwool’s experiences of MHCUs are focussed on behavioural disturbance and serious mental disorders in a context of severely limited discussion of the crucial role of support and interventions for people with common mental disorders, which form a substantial part of the burden of mental disorders in health services globally. There are also possible knock-on effects to what seems from Cottonwool’s experiences, that is, a ‘sink or swim’ approach, where inexperienced medical personnel take on the responsibilities Cottonwool speaks of. Their experiences would be unlikely to encourage them to consider a future career in healthcare and mental healthcare provision in this same context. If Cottonwool’s experiences are similar to those of others, this has implications for the growing and maturing of a workforce well equipped to implement evidence-based mental health interventions at scale to our country.

Our focus on one doctor’s experience in this study is a limitation, which means that we cannot know how generalisable Cottonwool’s experiences are. Our aim with the presented work was not to produce generalisable data, but rather to explore and report on the issues and concerns that young non-specialist doctors, like Cottonwool, may experience in providing mental health treatment at general healthcare level. However, we are able to say from the data from our larger project that the observations are in keeping with experiences of other healthcare personnel (e.g. social workers, and nurses), facility managers and patients, data which we will formally report on elsewhere. Cottonwool’s experiences are also similar to those reported by other clinicians that provide mental healthcare in a range of South African settings.^12,13^

## Conclusion

It is difficult to change health systems, an issue that is well documented,^14,15,16,17^ and we do not pretend to have the answer to the issues raised by Cottonwool. We believe, however, that a close-up account like this one is important, as it highlights the personal consequences for individuals of being placed on the front line of a challenged and under-resourced system, and underscores the fact that investments in health service skills and continuity of service are essential if provision is to match the high ideals of policy.

## Appendix 1

**TABLE 1-A1:** The interview guide used for data collection.

No.	Interview questions
1.	Please tell me about yourself and your work responsibilities at this facility.
2.	How long have you been working as (job description given in Question 1) at this facility?
3.	What is your understanding about the umbrella term ‘mental healthcare’?
4.	If you think about the patients who use this facility, what are the main mental health issues that they bring to the facility?
5.	How common are these issues, and how are they usually dealt with?
6.	The next few questions focus specifically on your role in providing mental healthcare at this facility. Would you mind discussing them with me? How long has mental healthcare provision been part of your work responsibilities? Did your role in mental healthcare provision evolve in any way over the years? Please elaborate. Can you explain the different mental healthcare treatment modalities available to service users at this facility? Who else is responsible for the provision of mental healthcare at this facility? Can you explain why you say so? What are your experiences with regard to mental healthcare provision at this facility? What are the factors that make it easy or difficult for you to provide mental healthcare treatment here? Please elaborate why you say this? What do you think needs to change to improve the quality of mental healthcare provision at this facility, if any? Please explain your answer to me. What are the factors that are already in place that enhance the quality treatment mental healthcare at this facility? Please elaborate what makes you say so?
7.	Are there any factors outside/away from this facility that impact your ability to provide mental healthcare to service users? Please elaborate on how it impacts mental healthcare provision here.
8.	What does your colleagues or members of the community think about mental healthcare at the facility and in the area?
9.	What would you wish tell key role players (e.g. the district manager or president) about the status of mental healthcare in your area or at this facility?
10.	Please give me an example of a case where you felt you did well in helping a patient with a mental health problem.
11.	Please give me a case where you felt you did not do well in helping a patient with a mental health problem.
12.	Is there anything else that you wish to tell me about mental health treatment provision that I must know?
